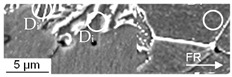# Load Direction-Dependent Influence of Forming-Induced Initial Damage on the Fatigue Performance of 16MnCrS5 Steel

**DOI:** 10.3390/ma13122680

**Published:** 2020-06-12

**Authors:** Kerstin Moehring, Frank Walther

**Affiliations:** Department of Materials Test Engineering (WPT), TU Dortmund University, Baroper Str. 303, D-44227 Dortmund, Germany; frank.walther@tu-dortmund.de

**Keywords:** fatigue performance, anisotropy, uniaxial load paths, pre-deformation, cold forward rod extrusion, initial ductile damage, case hardening steel 16MnCrS5, under eutectoid ferrite–pearlite

## Abstract

Forming processes influence the mechanical properties of manufactured workpieces in general and by means of forming-induced initial damage in particular. The effect of the latter on performance capability is the underlying research aspect for the investigations conducted. In order to address this aspect, fatigue tests under compressive, tensile and compressive-tensile loads were set-up with discrete block-by-block increased amplitudes and constant amplitudes, and performed up to fracture or distinct lifetimes. Aiming at the correlation of the macroscale mechanical testing results at the mesoscale, intensive metallographic investigations of cross-sections using the microscopical methods of secondary electron analysis, energy dispersive spectroscopy and electron backscatter diffraction were performed. Thereby, the correlation of forming-induced initial damage and fatigue performance was determined, the relevance of compressive loads for the cyclic damage evolution was shown, and material anisotropy under compressive loads was indicated. Finally, the need was addressed to perform further investigations regarding crack propagations and crack arrest investigations in order to clarify the mechanism by which initial damage affects cyclic damage evolution. The relevance of the principal stress axis relative to the extrusion direction was emphasized and used as the basis of an argument for investigations under load paths with different stress directions.

## 1. Introduction

Machine and plant engineering is the main pillar of the German investment goods industry and, due to the export quota of more than 78% in 2017, flagship worldwide. For providing goods for a variety of technical applications, a variety of components is necessary to fulfil the functional requirements. Thereby, the design is tailored to the expectable load types and profiles, the functionality and the required material properties. Despite the wider ranging design possibilities of newly developing production technologies [1], conventional production technologies remain the preferred technologies due to the capability for mass production, the material integrity and well-established methods to define the material properties. The latter is particularly true for forming technologies and, in particular, the forming technology of cold extrusion due to the possibilities to set the properties in the entire volume. Moreover, the technology of cold forward rod extrusion, which was used as the underlying technology for the material investigated in this study, also gives the possibility to set the material properties locally.

Realized by the selection of the forming parameters, the resulting stress state determines the profile of the plastic flow and thereby the material properties within forward rod extrusion. As a side-effect—besides changes in the hardness degree, the stress state and the general microstructure—ductile initial damage up to Chevron cracks can be induced [2]. Referring to this ductile damage as initial damage in this study, the average size of initial damage considered within this study was approximately 5 µm and, thus, small-size porosity or defectiveness. The effect of defects or pores on this length scale has not been addressed in the current state of research. The studies of Murakami or Baretta related to material defects considered only defects of different origins at a length scale of more than 40 µm [3,4,5]. The correlation with forming conditions and mechanical properties was firstly shown by Tekkaya et al. [6]. Investigating the technology of cold forward rod extrusion, earlier studies [7], that were only correlating the forming conditions and the material performance under service load conditions, were extended by Tekkaya et al. [6], who identified forming-induced initial damage to be the explanation for the correlation. Apart from this, the correlation of induced pre-deformation under different loading conditions with the fatigue performance has been addressed by [8] for TWIP-steel and [9] for DP600. Thereby, the relevance of the consideration of the load direction-dependent material behavior induced by pre-deformation arose, which has not yet been done for the case hardening steel 16MnCrS5 being processed using cold forward rod extrusion and being investigated in this study. The influence of the changes in the material properties needs to be considered for component design and lifetime prediction. The need for effect quantification is apparent regarding the dependence on the load case and the load direction. This is of particularly high importance due to the variety of mechanical load cases in existence under the variety of technical applications of products and workpieces processed using forming technologies and the small-sized nature of the initial damage investigated in this study.

As used for shafts, rigid shafts and gears, cyclic load paths and the understanding of the underlying mechanisms are of particular importance in order to establish safety factors as design criteria and to guarantee the functionality of gear units or power trains. During these applications, the joined components are subtracted to multiple loads like torsion, bending, tension or compression. Whereas the effect on the former three has been extensively investigated for decades and is considered by design criteria, this does not apply to compression loads to the same extent. On the contrary, ASTM E647-15e1 even pointed out to consider only the positive half cycle during the tension–compression load path for fatigue crack growth calculations [10]. The underlying assumption for this limitation was that compression loads increase the fatigue life by hindering the separation of planes (e.g., mode I crack propagation) and/or crack face closure [11] and are not critical for cyclic component failure. The contrary finding, viz. the relevance of compression loads for the damage evolution, has been shown throughout the last decades.

In consideration of the initial damage on a length scale below 10 µm, the relevance of crack growth considerations arises in the context of forming-induced initial damage. In representation as loci of increased stress concentration, initial damage as pores can be regarded as loci for crack nucleation and growth. Thereby, the effect of the reduced defect size in comparison to available studies with defect sizes of at least 40 µm/200 µm or √area > 200 µm [4,5] was not investigated. In [4], Murakami et al. characterized the influence of artificially using-pre-loading-technique generated surface cracks at a length scale of 200 to 1000 µm under torsional fatigue and rotation bending for medium carbon steels with a ferrite–pearlite, banded microstructure. Thereby, the negative dependence of the fatigue limit on the crack size was shown. Neither a conclusion at smaller length scales nor the influence of defects in the volume of the material or axial load direction was considered. The √area parameter model was used for the quantitative description and prediction of the fatigue lifetime, whereby the superimposing effect of hardness was indicated [4].

With regard to crack growth, the importance of compressive loads for material behavior has been shown throughout the literature under crack growth considerations for the load cases of fully compressive tests, compressive underloads and tension–compression tests [11]. However, the studies focus pre-dominantly on hexagonal magnesium alloys or cubic face-centered aluminum alloys. Cubic body-centered materials like the investigated case hardening steel 16MnCrS5 were not addressed. Under consideration of the clear material dependency of the crack growth mechanisms as emphasized by [12] and [13], a basic overview of the different findings is given hereafter. Pommier et al. related load history effects on the crack growth back to the occurrence of cyclic kinematic hardening and the Bauschinger effect [14]. Carlson and Kardomateas traced the material dependence back to compression-induced closure changes related to microstructural features and deformation properties [15]. Silva et al. emphasized the importance of compressive loads, especially for long cracks [11], and indicated compressive load-induced tensile residual stress fields emerging at the crack tip as the reason for these findings [11]. In accordance, [16], for ductile materials, and [17] have shown that cracks initiate and grow up to a certain length due to a tensile residual stress field produced by the cyclic loading at the initial crack tip position. Tack et al. indicated a higher crack growth rate for negative stress ratios *R* in comparison to the positive stress ratio of *R* = 0.1 and discussed the saturation phenomena regarding compressive effects [18]. Further investigations were conducted with respect to compression loads applied while shearing under torsional loading conditions. Doing so, the extension of coplanar crack growth was indicated as the underlying mechanism, whereby the growth rate was found to continuously decrease up to the bifurcation of the cracks [19]. Said et al. highlighted the crack propagation up to failure for negative stress intensity factors by mode shear mechanisms under compressive load during fretting fatigue [20]. The authors in [21] showed the effect of compression load on tension–compression fatigue after single-overload at negative stress ratios. Those in [22] highlighted the amplitude dependence of the fatigue life strength for bearing steels and related it back to the influence of surface residual compressive stresses. The authors in [23] indicated anisotropic material behavior for AZ80 magnesium alloy as well as the effect of forging for a forged and/and only extruded AZ80 Mg magnesium alloy using proportional and non-proportional axial–torsional fatigue testing methods. Toscano et al. showed tension–compression symmetry and strain amplitude dependence for casted, in comparison to forged, magnesium alloys and related this to the random texture of the former [24]. Additionally, they showed the diminishing influence of intermetallic inclusions and surface pores on compression–tension fatigue behavior [24]. Investigating concrete, Vicente et al. showed a negative correlation between porosity and fatigue, whereby small pores were less critical [25].

To sum up, the influence of initial damage on the mechanical properties in dependence on the load direction in terms of different types of loads applied in the direction longitudinal to the direction of forward rod extrusion as well as in terms of the loads applied longitudinal and transversal to the direction of forward rod extrusion is not addressed in current research studies. To meet this shortcoming, the current study contributes with a quantification of the influence of forming-induced initial damage in terms of the number of cycles to failure and the material reaction resulting under applied defined loads by comparing to material states that only differ with regard to forward rod extrusion-induced initial damage. The second contribution of this study to that shortcoming is the characterization of the underlying mechanisms under particular consideration of the mechanisms present under the applied compressive loads. In order to realize these aims, the microstructural material state including the state of initial damage is characterized. For the quantification of the effect of forming-induced initial damage, fatigue tests were conducted and compared. In order to identify the mechanisms, a microstructural characterization of the material states at distinct levels within the process of cyclic damage evolution was done, using intermittent fatigue tests and microscopic devices. To address the load direction dependency in terms of loads applied longitudinal and transversal to the direction of forward rod extrusion, cyclic compression fatigue tests were conducted with specimens extracted longitudinal and transversal to the direction of forward rod extrusion. The compressive load path was thereby selected in order to show the effect of compressive loads on the cyclic damage evolution and to get a first impression of the effect of compressive loads superimposed on other loads in complex, superimposed load paths like tension–compression or compression–torsion. The load direction dependency with regard to the load direction for axial loads being applied merely longitudinal to the direction of forward rod extrusion was characterized by performing cyclic compression, tension and compression–tension tests at load ratios of *R* = 10, *R* = 0.1 and *R* = −1, respectively, with specimens extracted in the direction of forward rod extrusion out of the workpieces. In addition to the two extruded material states, the as-received material state of the case hardening steel 16MnCrS5 before forward rod extrusion was analyzed in order to anticipate the forming influence on cyclic damage evolution and the basic load direction dependence of the material.

Proceeding in this way, the aim is to show a first implication of the load path dependency and the contribution of forming-induced initial damage to the anisotropy in the material behavior. Based on the results, conclusions shall be drawn as to further investigations needed to clarify the mechanism and interdependencies of cyclic damage evolution with respect to initial damage. Moreover, scientific knowledge for tests under more complex loading conditions with regard to load path and supporting investigations shall be generated and transferred to modeling and simulation in the long term.

## 2. Materials and Methods

### 2.1. Material and Material States

The investigations conducted were based on the low-alloyed case hardening steel 16MnCrS5 (1.7139, AISI 5117). The general chemical composition provided by the material supplier Georgsmarienhuette is listed in Table 1. The material was delivered as bars with diameter *d_ini_* = 40 mm and length *l_ini_* = 750 mm in a rolled and drawn, ferrite–pearlite annealed state (+FP). No additional heat treatment with regard to case hardening was undertaken.

Three different material states were investigated: the material state as-received (AR) and the two forward rod extruded material states. The latter were extruded out of the as-received material by using the forming parameters *ε_ex_* = 0.5, *2α* = 30° (E.530) and *ε_ex_* = 0.5, *2α* = 90° (E.590), with extrusion strain *ε_ex_* and shoulder opening angle *2α*. Within a radius *r*_hes,r_ = 4 mm, the effective strain was homogeneous [27]. The variation in the shoulder opening angle 2*α* did not affect the strain within *r*_hes,r_, because of flow-curve saturation, but induced a higher stress state for *2α* = 90°. Due to the increase in the maximum tensile stress component, the initial damage was increased. The stress state and thereby the degree of initial damage can be considered as homogeneous in a radius *r*_hss,r_ = 6 mm around the central axis of the rotationally symmetric workpiece and in the shaft in the axial direction. Based on the same extrusion strain, the hardness is comparably high for both extrusion states [27]. The residual stress state after specimen extraction out of the workpiece was negligible [25,28]. For further details regarding the material states after extrusion, refer to the investigations of [27].

### 2.2. Methods for Material State Characterization

In order to characterize the influence of forming-induced initial damage, the methods as described hereafter were used. The purpose of usage is additionally addressed. The devices used for the investigations are given.

The initial state, as well as the material states after distinct cyclic loading cycles, has been characterized using the methodology of scanning electron microscopy (SEM) using secondary electron (SE) analysis, energy dispersive x-ray spectroscopy (EDS) and electron backscatter diffraction (EBSD). The EBSD method was used for the identification of the forming-induced change of material properties, whereby the EDS method was used for the identification of the initial damage state. The quantification of this initial damage state with regard to the absolute area of pores was conducted using a grey value and EDS- based particle counting methodology (PCM). The analysis of the residual stress state (RSA) was conducted using x-ray diffractometry (XRD) in order to address the potential influence of forming that might superimpose the effect of initial damage. Hardness tests (HT) were performed in order to refute a superimposed influence of forming-induced hardening.

The SEM analyses were performed using a scanning electron microscope (Mira 3 XMU, Tescan, Brno, Czech Republic), equipped with an EDS-detector (Octane Pro, EDAX/Ametek, Berwyn, PA, USA) and an EBSD-detector (DigiView 5, EDAX/Ametek, Berwyn, PA, USA) for EDS and EBSD analysis, respectively. The PCM analyses were carried out using the commercial PCM genesis software (EDAX Particle Analysis, Genesis 3.2, EDAX/Ametek, Berwyn, PA, USA) on polished cross-sections using a gray value-based algorithm for the identification of surface inhomogeneities, combined with an EDS analysis to distinguish between pores and inclusions. A further explanation is given in [28]. The SEM analyses were conducted for the characterization of the state of cyclic damage evolution on the surface as well as for the microstructural characterization of the damage state in the material volume. For the latter, cross-sections were prepared longitudinal to the direction of the ferrite–pearlite microstructure and thereby to the direction of forward rod extrusion. In order to obtain a surface quality appropriate for state characterization, the material was ground down from P320 down to P2500 grade and polished with diamond suspensions of 6 µm, 3 µm and 1 µm. The surface was finish-polished with an active oxide polishing suspension with colloidal silica (SiO_2_) abrasives (OP-S suspension). In order to show the phase dependency of the damage evolution, the cross-sections as well as the surface were etched with Nital 3%. The residual stress state at the surface of the tested specimens was analyzed by using an x-ray diffractometer (D8 Discover, Bruker, Billerica, MA, USA) with a copper x-ray tube in side inclination and Bragg–Brentano geometry with sin^2^ψ-methode. The hardness of the material was characterized using a Shimadzu HMV-G hardness testing system (HMV-G, Shimadzu, Kyoto, Japan) with a Vickers indenter and an indentation force *F_ind_* = 4.903 N (HV0.5).

### 2.3. Methods for Characterization of Mechanical Properties

All experimental investigations were performed using the servo-hydraulic axial-torsional testing system (Walter + Bai, LFV-T250 T2500 HH, Löhningen, Switzerland) with *F_n_* = 50 kN and *M_t_* = 100 Nm nominal axial and torsional loads. The testing set-up varied for the cyclic compression and for the cyclic compression–tension as well as the cyclic tension investigations as detailed hereafter. In order to characterize the damage evolution, continuous tests up to failure as well as interrupted tests were performed (compare Section 2.3.1 and Section 2.3.2). The latter methodology is referred to as the intermittent testing method, ITM.

#### 2.3.1. Cyclic Compression Tests: Testing Set-up and Procedure

The experimental set-up was according to Figure 3 (RT) in Ref. [29]. The testing frequency was *f* = 1 Hz. The load ratio of *R* = 10 was applied by triangular endurance load cycles. The tests were conducted force-controlled. Referring to DIN EN ISO 50106 [30], pressure plates were modified with tungsten carbide plates as the contacting material for the specimen in order to prevent the plastic deformation of the compression device. The specimens were greased in order to reduce friction prior to testing.

Tests with continuous constant (constant amplitude test, CAT with *F_min_* = −0.6 kN and *F_max_* = −0.06 kN) and with block-wise discrete increased amplitude (load increase test, LIT with *F_a,start(R10)_* = −0.09 kN and ∆F*_a(R10)_* = −0.045 kN) were performed for up to 8000 cycles. A block was defined with 1000 cycles. The latter were performed to investigate the cyclic performance according to [31] and, thereby, the amplitude dependency of the mechanical behavior. The first were conducted as lifetime tests. The tests at constant amplitude were performed up to a run-out lifetime of 100,000 cycles. After run-out, the mesoscopic characterization of the material state was performed according to a successive testing methodology.

**Figure 1:**
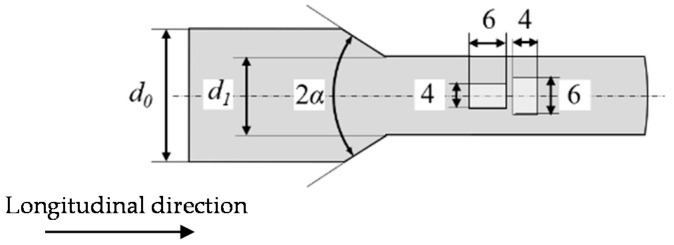
Schematic visualization of specimens used for cyclic compression tests, and extraction position. All dimensions are in mm.

The specimens were machined out of the bar material and the cold forward rod extruded workpieces, via electrical discharge machining (EDM). Due to the geometric limitations of the workpieces, cylindric specimens with a height to diameter ratio *h_0_/d_0_* of 1.5 (6 mm/4 mm, see Figure 1) were used, extracted from the center axis of the work piece in the axial (longitudinal, *l*) and radial (transversal, *t*) directions. The *l*-states are consequently orientated in and the *t*-states transverse to the fiber direction induced by the plastic material flow during forming. The term center axis refers to the geometrical center axis and an area around it with a radius *r* of 4 mm, in which the degree of initial damage can be considered as uniform according to [28] due to a uniform degree of strain during forming [6]. The further specimen preparation was performed according to [30], including grinding up to P1200 and polishing with diamond suspensions up to 1 µm, resulting in a surface roughness of the face surfaces of *R_z_* = 1.3 µm.

#### 2.3.2. Cyclic Tension and Compression–Tension Tests: Testing Set-up and Procedure

**Figure 2:**
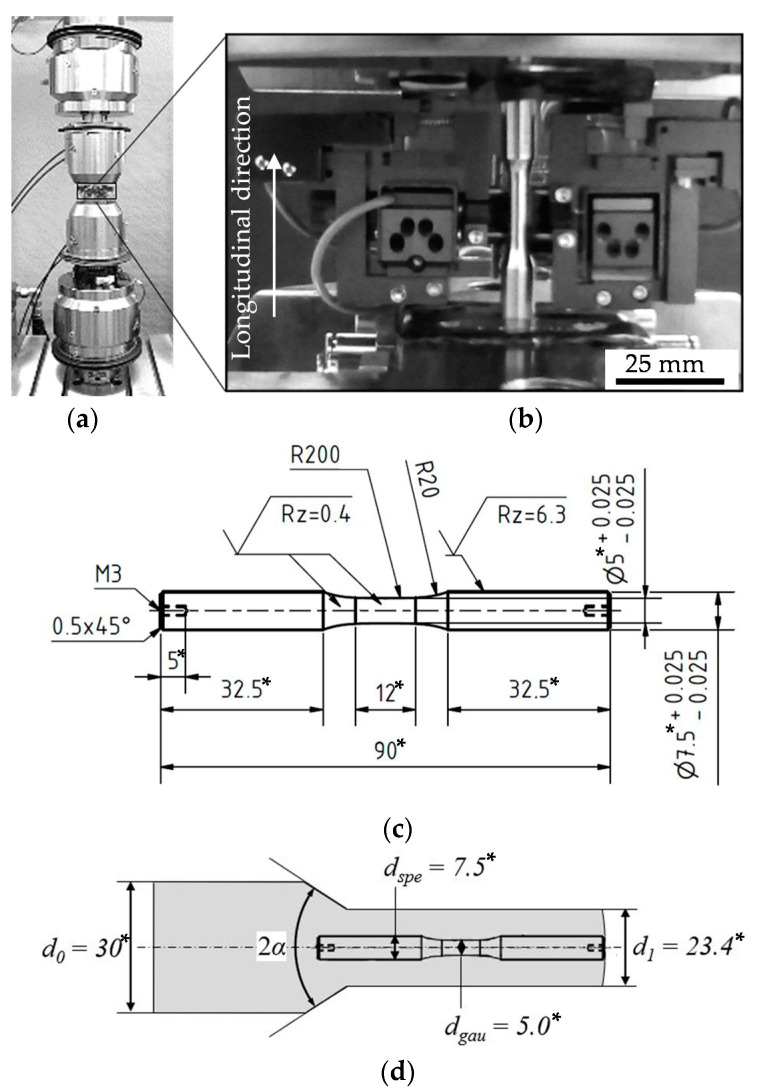
(**a**) Testing system Walter + Bai LFV-T250 T2500 HH; (**b**) testing set-up with specimen and axial extensometer with gauge length *l_gau_* = 10 mm used for cyclic tension (*R* = 0.1) and tension–compression tests (*R* = −1); (**c**) specimen for fatigue testing at load ratios *R* = 0.1 and *R* = −1; (**d**) Schematic visualization of extraction position. All dimensions are in mm*.

The experimental set-up is shown in Figure 2a,b. The testing frequency was *f* = 1 Hz. The load ratios were *R* = 0.1 for the cyclic tension tests and *R* = −1 for the cyclic tension–compression tests, respectively. The tests were performed load-controlled with triangular endurance load cycles. The tests were conducted with a block-wise discrete increased amplitude (load increase test, LIT) and for a selected load amplitude as constant amplitude tests (CAT).

The maximum load during the constant amplitude tests was *F_max_* = 6 kN for the tests performed at *R* = 0.1 and *R* = −1. The constant amplitude tests were interrupted at 100,000 cycles for the characterization of the damage state. After interruption, mesoscopic characterization of the material state was performed according to a successive testing methodology (intermittent testing method, ITM). The results of the tests at *R* = 0.1 and *R* = −1 presented in this study focus on the meso-structural differences with regard to the phenomenological cyclic damage evolution in the test conducted at *R* = 10 (see Section 2.3.1). The load increase tests (LIT) were conducted up to failure, whereby one block consisted of 10,000 cycles. The starting amplitudes for the tests at load ratios *R* = 0.1 and for *R* = −1 were *F_a,start(R01)_* = 2.5 kN and *F_a,start(R-1)_* = 1.95 kN, respectively. The loads were increased block-wise by *∆F_a(R01)_* = 0.5 kN and *∆F_a(R-1)_* = 0.4 kN, respectively.

The specimens were machined out of the bar material and the cold forward rod extruded workpieces in axial direction, viz. longitudinal direction, l, via turning. The specimen geometry (see Figure 2c) and extraction position (see Figure 2d) were selected according to [6]. The surface of the specimen was ground from P320 down to P2500 grade and polished with diamond suspensions of 6 µm, 3 µm and 1 µm. The obtained surface roughness was R_a_ = 0.59 µm.

## 3. Results

### 3.1. Material State

**Figure 3:**
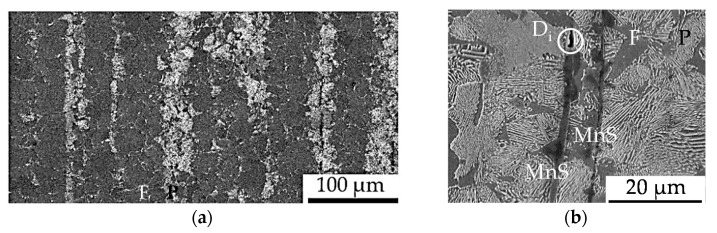
(**a**) Ferrite (F)–pearlite (P) microstructure after etching with Nital 3%; (**b**) forming-induced initial damage (D_i_) at the tip of a manganese sulfide (MnS)-inclusions in pre-dominantly pearlite (P) grains interrupted by ferrite grains (F).

The initial state of the material was characterized by means of the fatigue life time relevant material criteria of phase distribution and phase orientation, grain size and grain orientation as well as morphology of the initial damage and orientation of the initial damage as follows. The underlying aim was the characterization of the influence of forming by forward rod extrusion with respect to the general microstructure, the residual stress and hardness state and the initial damage state. The residual stresses and hardness were shown in accordance with the results presented in [2,27,28] to be comparable for the extruded material states. The hardness levels were quantified with 139HV0.5 ± 10 (AR) and 158 HV0.5 ± 7 (E.530/E.590), respectively. The residual stress states at the specimen surface were in consideration of the comparable measurement tolerance and in the range of zero for the three material states investigated. A typical ferrite–pearlite banded structure was shown microscopically in the rolling (AR) and extrusion direction (E.530/E.590) after transverse and longitudinal cross-section preparation followed by etching with Nital 3% (Figure 3a).

**Figure 4:**
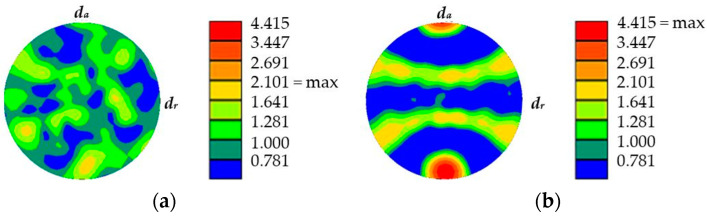
Electron backscatter diffraction (EBSD) texture plot of the longitudinal cross-sections in the flow direction for the material states (**a**) as-received (AR) and (**b**) after extrusion (E.530/E.590) with radial direction *d_r_* and axial direction *d_a_*. The maximum value of the texture indicated for the different material states is indicated with *max*.

The extruded material states (E.530/E.590) showed an elongation of grains, resulting in a length to width ratio *r_l/w_* of 4 compared to *r_l/w_* = 2.3 for the as-received (AR) material state. The average grain size was the intercept length grain size. The material states E.530/E.590 showed comparable average grain sizes. The grains were randomly distributed in the material state AR but preferentially orientated in the flow direction for the material states E.530/E.590. The corresponding textures with respect to the flow direction are indicated in Figure 4 in terms of pole figures for the cross-sections analyzed. The pole figure of material state AR indicates a random texture with no preferred orientation. On the contrary, a strong texture of the material states E.530/E.590 in the flow direction, viz. the axial direction *d_a_*, is depicted (Figure 4b). A statistically significant difference between the material states E.530/E.590 was not detectable.

In addition to the increasing pore volume from the state AR, over E.530 to E.590 as also shown by [28], a preferential orientation of the initial damage was shown by using the center axis of the individual pores, viz. the initial damage, in reference to the flow direction. The interfaces between the ferrite and pearlite grains as well as the entire pearlite phase were identified as the locations of non-metallic MnS precipitations (MnS-inclusions) as shown in Figure 3b. This finding is related to the decreased sulfide solubility with the reduced temperature during the solidification of the steel (MnS-precipitation) in combination with the time offset after solidification between the precipitation of ferrite and under-eutectoid pearlite formation (MnS-location) for this under-eutectoid steel. The manganese sulfides are well deformable, being extended longitudinal and compressed transversal to the extrusion direction in material state E.530/E.590 compared to AR.

As shown by Hering et al., the pore content as an indicator for initial damage detected by an electron dispersive x-ray spectroscopy (EDS)-based particle identification method [32] decreased from *A_in,d_* = 84 µm^2^ (E.530) to *A_in,d_* = 302 µm^2^ (E.590) [28]. The morphology of the initial damage for the state E.530/E.590 was characterized as documented in Table 2. The main type of initial damage with a partition of 95% was the decohesion of the interface between the metallic matrix and MnS-inclusions in this micro-alloyed steel. An exemplary morphology is shown in Table 2.

The pre-dominant location of this type of initial damage was the interface between the ferrite and pearlite grains or merely pearlite grains, respectively. The remaining 5% of the initial damage manifested in the representation of an interface decohesion of metallic matrix Al_2_O_3_ or other non-metallic inclusions in the ferrite phase or the decohesion of grain boundaries at pre-dominantly triple points as indicated in Table 2. The orientation of this initial damage was in the flow direction. The triaxiality-induced isotropic pore nucleation and growth initiated by manganese-sulfide breakage under load as well as the separation of the interface between metallic material matrix and inclusions became obvious as underlying mechanisms.

### 3.2. Mechanical Properties

#### 3.2.1. Cyclic Compression Tests and Microstructural Correlation

**Figure 5:**
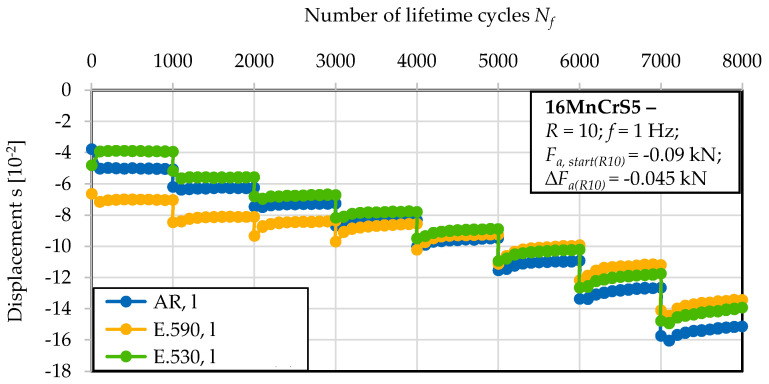
Material reaction under cyclic compression load conditions obtained longitudinal to, viz. in, the extrusion direction (l) after the load increase test (LIT).

The cyclic compression fatigue performance after loading in the longitudinal direction is indicated by the stress–lifetime (S–N) curves in Figure 5. The conducted test with the block-wise increased load amplitude (load increase test, LIT) gave rise to a cyclic damage accumulation-dependent effect of the initial damage. The material reaction in the initial state due to the applied compressive load amplitude implies an influence of the initial damage.

**Figure 6:**
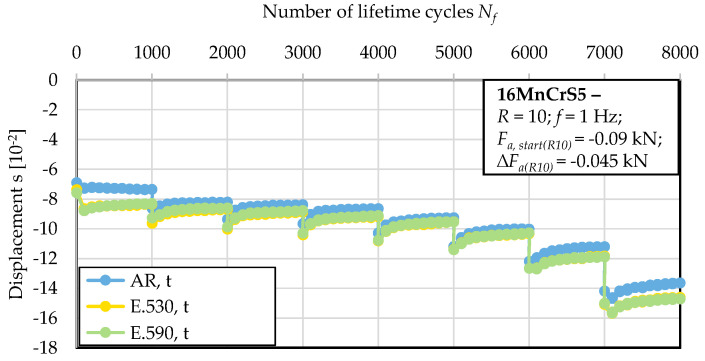
Material reaction under cyclic compression load conditions obtained transversal to the extrusion direction (t) after the load increase test (LIT).

The investigated material state E.530 with the lower degree of initial damage showed an approximately 75% higher residual compression force in reaction to the applied upsetting deformation compared to the material state E.590. Thereby, no indication of proceeding cyclic deformation with regard to the cyclic dislocation mechanisms arose, as indicated by the constant material reaction over the 1000 cycles of the first block. Within the thereafter following blocks under discrete increased load amplitudes, plastic deformation in the sense of a decreased stress-to-deformation ratio was observed. The softening ratio remained approximately constant within the individual blocks and independent of the initial state of damage. The effect of the initial damage was not detectable with respect to statistical significance for compression loading transversal to the direction of forward rod extrusion. Figure 6 indicates this in terms of a nearly equal material reaction in terms of the displacement of the three material states investigated under the applied loads. The resilience of the material was reduced by at least 25% for all the material states investigated compared to the specimen manufactured longitudinal to the direction of forward rod extrusion. Comparing the results of Figure 5 and Figure 6, the anisotropic behavior was consequently less pronounced for the less-initially damaged material state E.530. The material in the as-received material state, AR, showed a more pronounced anisotropy in the material reaction compared to the E.530/E.590 material states. A dependency on the loading direction with respect to the direction of forward rod extrusion became obvious. A reduced deformation capability was shown for cyclic compressive loading in the longitudinal direction. For the life-time orientated tests at a constant compression amplitude with a minimal load of *F_min_* = 6 kN, the S–N curve showed no distinct material reaction up to the run-out at 100,000 cycles for the extruded material states. Material state E.590 showed a worse performance than material state E.530 with regard to an increased material deformation under comparable loads.

**Figure 7:**
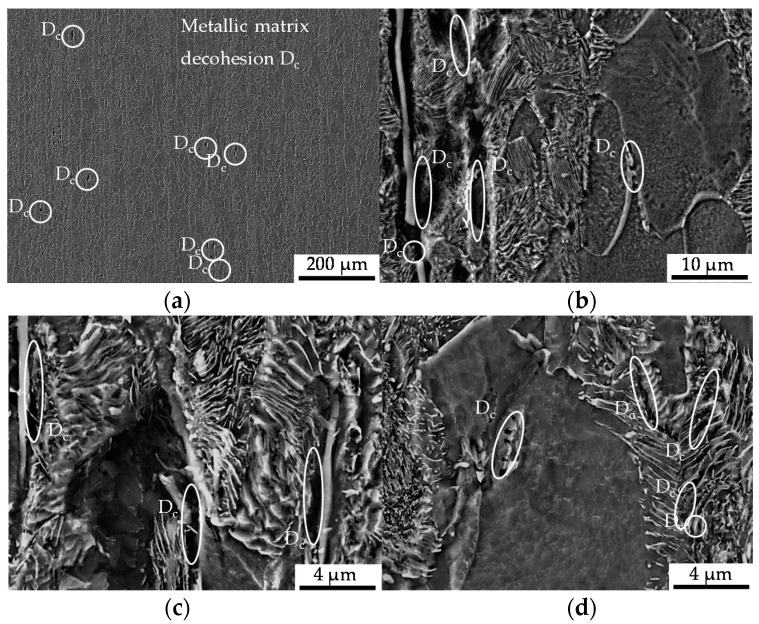
Cyclic damage (D_c_) induced under cyclic compression load paths: (**a**) overview of the ferrite–pearlite microstructure giving a representation of the quantity of cyclic load-induced cyclic damage in the form of metallic matrix decohesion and a subpicture of detailing; (**b**) detailed view of pore-coalescence at MnS-inclusions clustered in the pearlite phase (**left**), and grain boundary decohesion (**right**); (**c**) detailed view of the interface decohesion between the metallic matrix and MnS-inclusions; (**d**) Fe3C-cracking (**left**), and cracks grown in grains belonging to the pearlite material phase (**right**).

The microstructure induced by the constant amplitude tests is displayed in Figure 7 as obtained after the longitudinal sectioning of the fatigued sample after 100,000 cycles of the constant amplitude test (CAT). The state of damage in terms of initial and cyclic damage was found to be 10% higher for the material state E.590, whereby the initial difference was slightly increased. The material showed pre-dominantly decohesion effects between the material matrix and the non-metallic inclusions, viz. pores, around MnS-inclusions with a preferred location in the pearlite phase of the metallic structure as an indicator for cyclic damage. In terms of a dependence on the nearest distance between the inclusions, clusters of inclusions were determined to act as the location for advanced pore coalescence (see Figure 7). Besides that, the fracturing of MnS-inclusions and grain boundary cementite (iron carbide, Fe3C) and local ferrite grain boundary decohesion were occasionally observed. A further increase of cyclic damage evolution manifested as the appearance of cracks and holes in the pearlite phase or under the cementite slats.

**Figure 8:**
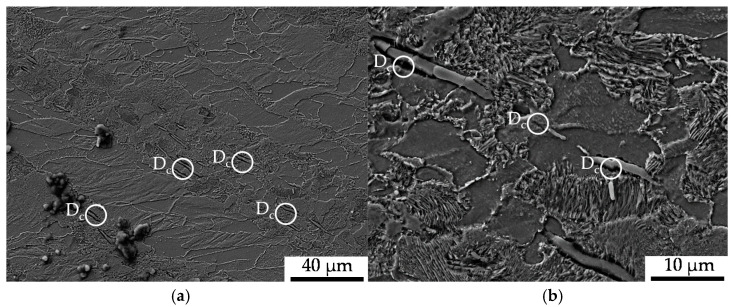
Cyclic damage (D_c_) induced under cyclic compression load paths in the bulged area of the material: (**a**) overview of the ferrite–pearlite microstructure; (**b**) detailed view of the interface decohesion between the metallic matrix and MnS-inclusions.

In the bulged area of the specimen, which is characterized by a non-uniform stress state due to bulging, the deformation of MnS-inclusions in the bulging direction accompanied by the decohesion of the metallic matrix and the MnS-inclusions decohesion was observed (see Figure 8). The grains belonging to the ferrite phase showed no mesoscopic signs of cyclic fatigue damage.

#### 3.2.2. Cyclic Tension Tests and Microstructural Correlation

**Figure 9:**
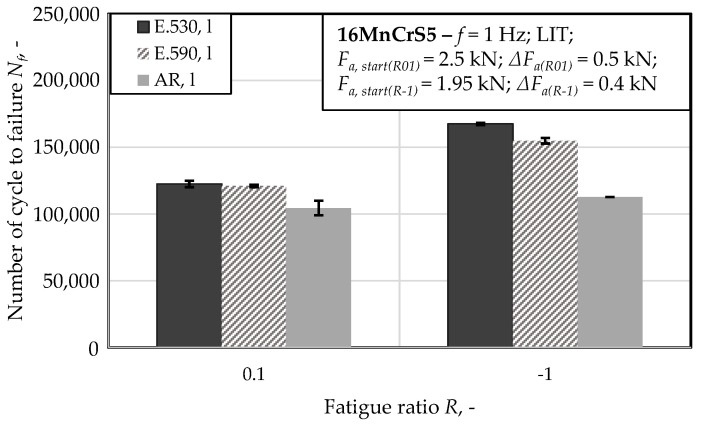
Material reaction under cyclic tension (*R* = 0.1) and cyclic compression–tension (*R* = −1) load conditions obtained longitudinal to, viz. in, the extrusion direction (l) after the load increase test (LIT).

**Figure 10:**
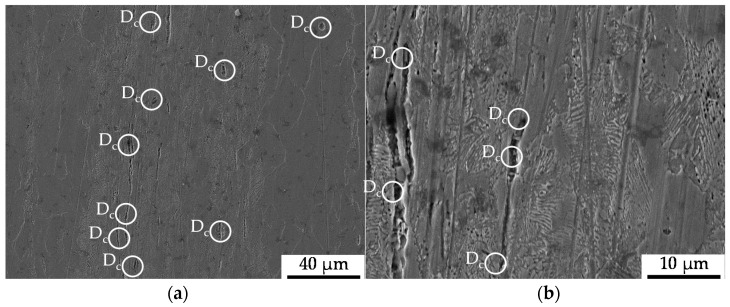
Cyclic damage (D_c_) induced under cyclic tension load paths: (**a**) overview of the ferrite–pearlite microstructure; (**b**) detailed view of interface decohesion between the metallic matrix and MnS-inclusions.

**Figure 11:**
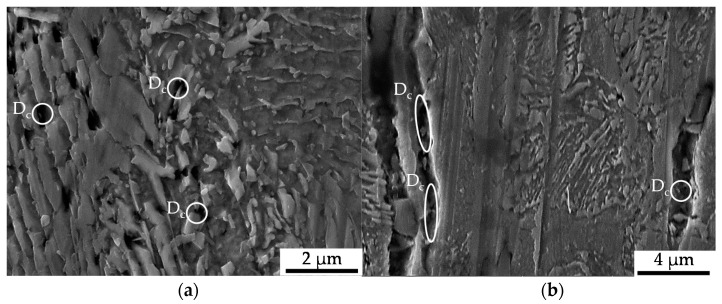
Cyclic damage (D_c_) induced under cyclic tension load paths at a higher magnification: (**a**) detailed view of void nucleation in the pearlite phase; (**b**) detailed view of the interface decohesion of the metallic matrix and MnS-inclusions.

The underlying tests for the results presented in this section were conducted to characterize the effect of the load direction dependence of the influence of forming-induced initial damage under loads applied longitudinal to the direction of forward rod extrusion in different directions. The investigations, conducted at load ratio *R* = 0.1, implied no statistically significant dependence of the cyclic performance on the initial damage (see Figure 9). A difference of 1% between the material states E.530 and E.590 with regard to the number of cycles to failure was detected, which is fairly within the scatter observed (compare Figure 9). Thus, no statistically significant difference between the material states E.530 and E.590 was detectable. The tension fatigue test induced the damage depicted in Figure 10 and Figure 11.

**Figure 12:**
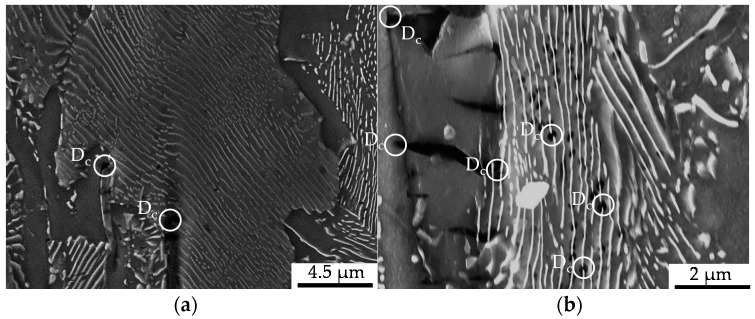
Cyclic damage (D_c_) induced under cyclic compression–tension load paths: (**a**) detailed view of isotropic crack growth at the tips of MnS-inclusions; (**b**) detailed view of MnS-inclusion assisted microcrack growth (**left**) and pore nucleation in the pearlite phase.

Compared to the compression fatigue tests, a more pronounced tendency for isotropic pore elongation, viz. crack growth, in the load and thereby extrusion direction occurred. The role of the MnS-inclusions, being fractured or representing the initiation points for the cracks, that cause the decohesion of the metallic matrix, remained very important. Withal, a higher partition of material volume is affected compared to that in the cyclic compression tests. This is due to the load-assisted crack growth in the longitudinal direction compared to the proceeding material degradation around the inclusions caused by pore growth in the radial direction under compressive loads (Figure 7). Void formation in the grains of the ferrite as well as the pearlite phase and at the interface between the two phases was observed to a higher extent under pure tensile loading, as depicted in Figure 12. These shown phenotypes of cyclic damage evolution in the volume occurred with the elongation of the grains in the longitudinal direction.

#### 3.2.3. Cyclic Tension–Compression Tests and Microstructural Correlation

The material states E.530 and E.590 performed differently under tension–compression load. The initial damage and the fatigue performance correlated in the way that the reduction of the degree of initial damage results in an increase in the cyclic performance.

**Figure 13:**
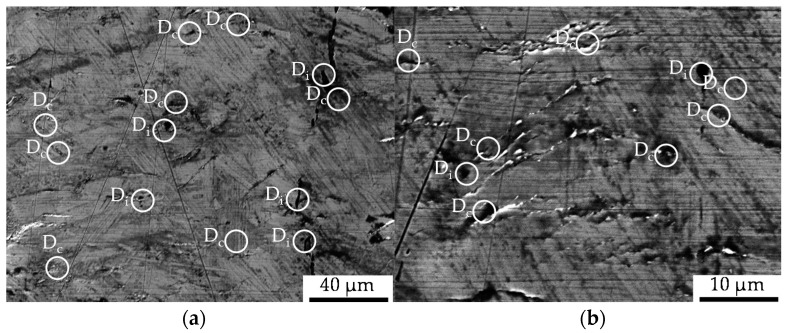
Cyclic damage (D_c_) on the surface of the specimen induced under cyclic compression–tension load paths interacting with the initial damage (D_i_): (**a**) initial interface decohesion of the metallic matrix and the MnS-inclusions identified after the intermittent testing at defined lifecycle phases interacting with shear bands; (**b**) decohesion of the initial interface matrix and the MnS-inclusions identified after intermittent testing at defined lifecycle phases interacting with intrusions and extrusions.

The cyclic damage was observed for, with regard to the investigations under compressive and tensile load amplitudes, comparable load amplitudes after 100,000 cycles. The manifestation of cyclic damage was in accordance with the results obtained for the compressive and tensile load cases, whereby it was superimposed and thereby less pronounced than in the corresponding pure compressive tensile load cases (see Figure 12). As under the compressive and tensile cyclic load conditions, the origin of macro-crack growth, which was limiting the life time, was on the outer surface. Thereby, the initial damage prior to testing was comparable with the initial damage in the volume, since the specimen was being manufactured out of the workpiece volume. Anyway, some MnS-inclusions were removed due to manufacturing and some surface roughness remained after polishing. The latter did not affect the cyclic damage mechanism significantly, as interrupted tests with microstructural surface characterization (intermittent testing method, ITM) showed. The initial damage was found to interact with the phenomena of uniaxial cyclic damage, viz. shear bands, intrusions and extrusions (see Figure 13), and micro-voids, nucleating and growing to the state of lifetime-limiting macro-cracks.

## 4. Discussion

The contribution to research of the present study was stated in Section 1 to be, firstly, the quantification of the effect of forming-induced initial damage in the dependence on the load direction by means of the number of cycles to failure and the resulting material reaction under applied defined loads. Secondly, the characterization of the underlying mechanisms was targeted, whereby it was highlighted that the initial material state needed to be firstly characterized in order to realize this aim. The results presented in Section 3 are discussed hereafter with regard to these two contributions in dependence on and correlation with each other. The material state after forward rod extrusion and the potential implications for the mechanisms are discussed prior to these in order to provide the discussion basis and are additionally considered in order to realize the two target contributions of this study.

The extrusion process gave rise to the development of initial damage, which has a preferred orientation in the direction of forward rod extrusion and thereby the direction of the ferrite–pearlite banded microstructure (Section 3.1 and Figure 4). Referring to the stress state during the extrusion of the work pieces dominated by positive hydrostatic stress with the dominance of the axial tensile component of the stress tensor [27], the preferred orientation is in good agreement. This also applies to the types of initial damage. As published by Rice et al., the stress-assisted plastic deformation promotes the nucleation and growth of micro-voids ([33], Figure 7, Figure 8 and Figure 10), whereby the shown central role of the non-metallic MnS-inclusions (Table 2 and Figure 7 and Figure 10) is also indicated by Zapara/Augenstein/Helm [34]. The MnS-inclusions are known to induce anisotropic material behavior [35] including the behavior under cyclic load sequences ([36], for forged and hardened medium carbon steel [37]). Alongside the texture of the extruded material and the fiber direction induced with the elongation of grains in the longitudinal direction and the ferrite–pearlite banded two-phase structure, the main microstructural sources of anisotropy are combined (Section 3.1, Table 2). Whereas the influence of the former is within the scatter of the material and for the material states E.530 and E.590, under the assumption of random distribution and variation, equally, the latter is comparable for both extruded states. The preferred orientation indicated by the pole figures can thereby be seen as an explanation for the anisotropy regarding the mechanical behavior in the longitudinal and transversal direction, but not for the differences between the material states E.530 and E.590. Consequently, the differences in the arising anisotropy, viz. load direction dependence, for the two extruded material states (Figure 5 and Figure 6) need to be related back to the deviations of anisotropy induced by the initial damage between the material states.

The influence of initial damage was quantified by means of the number of cycles to failure and the material reaction under comparable loads in dependence on the load direction. The finding, that the effect of forming-induced ductile initial damage is statistically not significant under compression fatigue at the load ratio of *R* = 10 with the principal stress axis transversal to the flow direction (Figure 6), can be discussed with respect to stress concentrations and microstructural barriers as underlying mechanisms. Considering classical crack growth theories, initial damage (pores), considered as crack nucleation loci, promotes the interface decohesion between the metallic matrix and the MnS-inclusions in the radial direction (Figure 7), due to compressive loads along the principal axis in the extrusion direction. On the contrary, the propagation might be hindered by microstructural barriers in the axial direction (Figure 10), due to compressive loads along the principal axis transversal to the extrusion direction. The microstructural barriers are represented by the fiber direction and especially the location of the pearlite grains, the MnS-inclusions elongated and the increased axial-length/radial-thickness ratio of the pores (Section 3.1) transversal to the operating compressive load. Because of these microstructural characteristics, the stresses would be reduced and the influence of initial damage hindered, respectively compensated according to the findings of [11] for crack growth analysis. In contrast to this, the stress concentration is increased due to loads along the principal axis longitudinal to the direction of forward rod extrusion. The propagation of the initial damage is manifested by the mechanism of an increased area of matrix/inclusion decohesion, as highlighted in Figure 7. Therefore, the effect of compressive loads on the influence of the initial damage cannot be denied. This is in agreement with [38]. The apparent contradiction with the results of Hering et al. obtained for the notch impact test (Charpy impact test [27]) can be resolved by considering the multiaxial stress stats induced during testing. The notch of the specimen was located in the radial direction [27], such that the impact load was induced transversal to the extrusion direction. The resulting stress state is fairly multiaxial, wherefore the microstructural barriers could be overcome and the influence of the initial damage could be consequently detectable.

Following this line of argumentation in combination with the findings of [11,39], the results obtained at load ratio *R* = 0.1 under pure cyclic tension (Section 3.2.2), the relevance of plastic properties needs to be discussed as an explanation for the load direction-induced decreased, up to eliminated influence of the initial damage with regard to the cycles to failure (Figure 9). Based on the argument of the reduced crack driving force as the underlying mechanism due to the missing compressive half-cycle [11], further investigations need to be performed. Thereby, the theory that the reduced crack driving force due to the lack of the compressive half-cycles hinders the influence of the initial damage, in the sense that the cyclic damage evolves fairly independently of the initial damage, should be investigated. The operating load amplitude must thereby be considered as a main determinant for the falsification or verification of this theory. The findings of [27], that quasi-static tensile tests are an adequate method for the detection of the influence of initial damage, are thereby not contradictable. This is apparent, because the high tensile forces could activate pore growth and crack propagation, whilst overcoming barriers, supported by material plasticity. Qian et al. have shown that for cast steels, shielding effects between pores interacting can arise and thereby compensate for the damaging effect of pores in a similar manner in dependence on the operating amplitude [40].

In comparison to the results for this cyclic tension load path, the results obtained under cyclic tension–compression loading (Section 3.2.3 and Figure 12) indicated a load-direction dependence. The influence of the initial damage correlated with an increased performance with regard to the number of cycles to failure and material reaction, positively. A reduced degree of initial damage resulted in an increased material performance for the tow material states E.530/E.590 investigated. This finding agreed well with the findings for forging under uniaxial and multiaxial load paths for magnesium alloys [23,24], for the casting of magnesium [41] and iron [42], and for additive manufacturing [43] with pores at a scale fairly above the 20 µm scale of the investigated initial damage. The dependence of the influence of the initial damage was quantified in comparison to the results obtained under the cyclic tensile load path with more than 30% according to Figure 9. The relevance of the compression half-cycle highlighted for the fully compressive loads implied relevance to the cyclic damage evolution for the load case of cyclic tension–compression in accordance with [11]. The results of the microstructural characterization support this in the way, that the phenomena detected for the single load cases are combined, even though weakened (compare Figure 7, Figure 10 and Figure 12), possibly due to the interdependencies between both load cases in the half-cycle sequences. The crack was initiated at the specimen surface, whereby the initial damage interfered within the cyclic damage evolution (e.g., the formation of slip bands, intrusions or extrusions). Thereby, the implied dependency of the influence of the initial damage on the surface condition, respectively the integrity (e.g., roughness, residual stress state or presence of stress raisers), might limit the transferability of the results. This is particularly true as different damage mechanisms might interact in different ways with the initial damage.

In summary, the effect of initial damage on the load direction dependence can be quantified in terms of a reduced number of cycles to failure and an increased material reaction under comparable loads for the two extruded material states E.530/E.590 investigated. The underlying mechanisms were discussed to be related to microstructurally affected enhanced material degradation under tension and compression load cycles due to initial damage-induced crack growth and decohesion effects within the metallic material matrix and phases as well as between the metallic matrix and the non-metallic MnS-inclusions.

## 5. Conclusions

The present study highlights the influence of cold forward rod extrusion-induced initial damage on the mechanical behavior of the case hardening steel 16MnCrS5 subjected to cyclic axial loads by comparison between the material states E.530/E.590. A load direction dependence was indicated and quantified. An effect of initial damage on the cyclic damage evolution in the sense of increased material degradation was shown for compressive and combined tensile-compressive load cases. This was particularly true for both the quantification of the influence of initial damage and the underlying mechanisms of the dependence on the load direction. The influence of initial damage vanished in cyclic tensile load cases. The role of compressive loads in the cyclic damage evolution was emphasized in terms of the mechanisms of a promoted matrix/inclusion-interface decohesion in the initially damaged material. Thereby, the influence of the entire forming-induced microstructure with regard to the direction of forward rod extrusion was shown. Interdependencies of the effect of the general microstructure in terms of the preferred grain and phase orientation and the initial damage gave rise to the load direction dependence investigated. The load direction dependence was shown with regard to the loading direction relative to the direction of forward rod extrusion and the different load paths in the longitudinal direction investigated. A contribution of the initial damage to this mechanical anisotropy of the material was shown in terms of enhanced material degradation due to isotropic crack growth in the longitudinal direction to the direction of forward rod extrusion as an underlying mechanism. The relevance of the intermittent testing procedure in addition to the macroscale mechanical testing for the objective of mesoscale effect correlation became obvious. Further investigations are necessary in order to separate the influence of initial damage and the mechanisms and interdependencies of the initial damage contributing to the cyclic damage evolution in dependence on the load direction.

The results of this study are in general agreement with general findings in the literature. Due to the fact that the current state of the art is limited either to fairly higher degrees of porosity—like casting, melting or forging defects—or to macroscale approaches to the investigation of the correlation of pre-deformation and fatigue performance, the comparison and discussion was phenomenological. The implications of the type of forming or manufacturing (additive manufacturing) must be considered whilst conducting further investigations and discussing the results comparatively. To date, there is no consensus on the mechanism and determinants of the effect of forming-induced initial damage on the cyclic damage evolution.

The need arises to conduct further investigations with regard to the arrest of microstructurally short cracks nucleated at pores, viz. initial damage, in order to clarify the mechanism of the interdependencies of the initial damage and cyclic damage evolution. Thereby the via extrusion formed entire metallic material structure needs to be considered. Based on this, the load direction dependence of the influence of the initial damage in terms of crack propagation and crack closure stress effects needs to be investigated further. Additionally, the influence of the mean stress effect in comparison to the effect of stress amplitude must be scientifically addressed, in order to identify the interdependencies of initial damage and fatigue performance.

## Figures and Tables

**Table 1:** Chemical composition of the investigated low-alloyed case hardening steel 16MnCrS5, in wt % [26].

Material	C	Si	Mn	S	Cr
16MnCrS5	0.14–0.19	≤0.4	1.0–1.3	≤0.02–0.04	0.8–1.1

**Table 2:** Types, location and orientation of forming-induced initial damage (D_i_) in the material states E.530/E.590 with respect to the fiber direction after extrusion (FR), with the decohesion of the interface between metallic matrix and MnS-inclusions as the main type of initial damage (>95%).

Type	Position	Orientation	Exemplary Morphology
Matrix decohesion at interface with MnS-inclusions	Interface of ferrite/pearlite or pearlite grains	Longitudinal (flow direction)	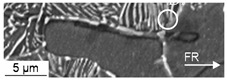
Matrix decohesion at intermetallic inclusions	Ferrite grains	Longitudinal (flow direction)	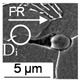
Matrix decohesion at grains, grain boundaries, triple points	Ferrite phase	Undefined